# Crystal structure of (*S*)-2-amino-2-methyl­succinic acid

**DOI:** 10.1107/S2056989015016709

**Published:** 2015-09-12

**Authors:** Isao Fujii

**Affiliations:** a School of Science, Tokai University, 4-1-1 Kitakaname, Hiratuka, Kanagawa 259-1292, Japan

**Keywords:** crystal structure, succinic acid, zwitterion, hydrogen bonding, three-dimensional framework

## Abstract

The title compound, C_5_H_9_NO_4_, crystallized as a zwitterion. There is an intra­molecular N—H⋯O hydrogen bond involving the *trans*-succinic acid and the ammonium group, forming an *S*(6) ring motif. In the crystal, mol­ecules are linked by O—H⋯O hydrogen bonds, forming *C*(7) chains along the *c*-axis direction. The chains are linked by N—H⋯O and C—H⋯O hydrogen bonds, forming sheets parallel to the *bc* plane. Further N—H⋯O hydrogen bonds link the sheets to form a three-dimensional framework.

## Related literature   

For general background and biological properties of 2-methyl­aspartic acid (MeASP), see: Pfeiffer & Heinrich (1936 ▸); Delbaere *et al.* (1989 ▸); Nobe *et al.* (1998 ▸). For the absolute configuration and synthesis of the title compound, see: Terashima *et al.* (1966 ▸). For the crystal structure of related racemic compounds, see: Derricott *et al.* (1979 ▸); Brewer *et al.* (2013 ▸). For the crystal structure of dl-ASP, see: Flaig *et al.* (1998 ▸).
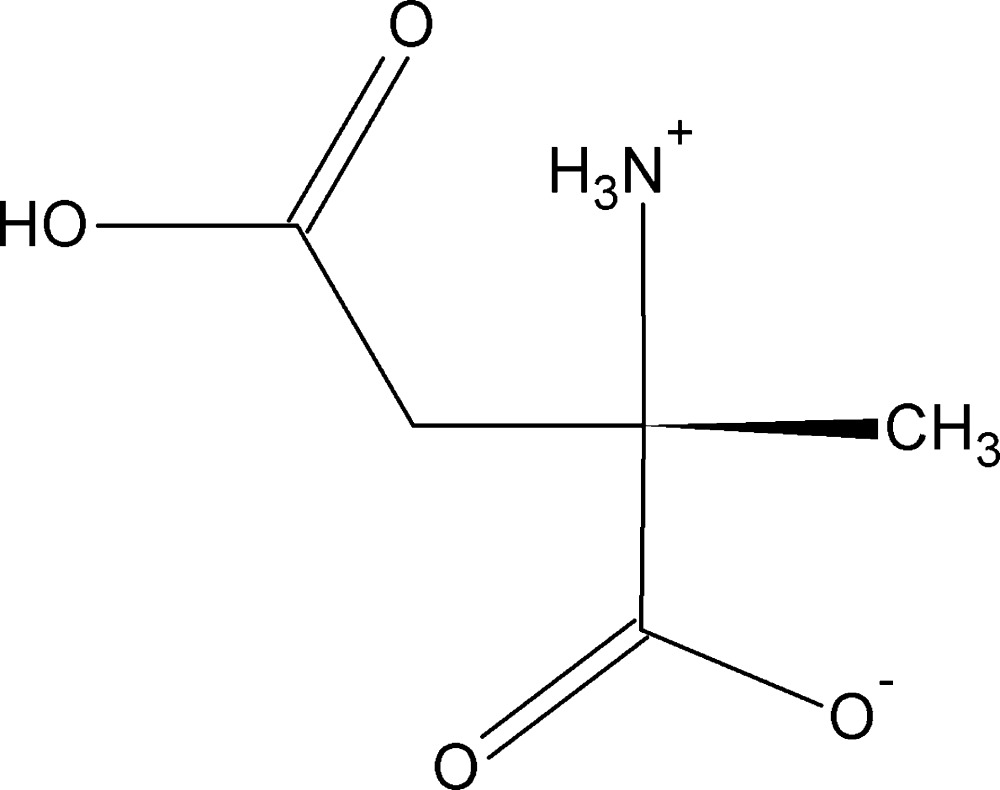


## Experimental   

### Crystal data  

C_5_H_9_NO_4_
*M* *_r_* = 147.13
Monoclinic, 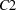

*a* = 8.3398 (12) Å
*b* = 9.6725 (10) Å
*c* = 8.0671 (10) Å
β = 95.175 (5)°
*V* = 648.09 (14) Å^3^
*Z* = 4
Cu *K*α radiation
μ = 1.14 mm^−1^
*T* = 297 K
0.4 × 0.2 × 0.2 mm

### Data collection  

Enraf–Nonius CAD-4 diffractometer
Absorption correction: ψ scan (North *et al.*, 1968 ▸) *T*_min_ = 0.76, *T*_max_ = 0.81
843 measured reflections
700 independent reflections
699 reflections with *I* > 2σ(*I*)
*R*_int_ = 0.019
3 standard reflections every 300 reflections intensity decay: none

### Refinement  

*R*[*F* ^2^ > 2σ(*F* ^2^)] = 0.034
*wR*(*F* ^2^) = 0.096
*S* = 1.27
700 reflections
109 parameters
2 restraints
H atoms treated by a mixture of independent and constrained refinement
Δρ_max_ = 0.29 e Å^−3^
Δρ_min_ = −0.21 e Å^−3^

### 

Data collection: *CAD-4 Software* (Enraf–Nonius, 1989 ▸); cell refinement: *CAD-4 Software*; data reduction: *XCAD4* (Harms & Wocadlo, 1995 ▸); program(s) used to solve structure: *SHELXS97* (Sheldrick, 2008 ▸); program(s) used to refine structure: *SHELXL2014* (Sheldrick, 2015 ▸); molecular graphics: *ORTEP-3* for Windows (Farrugia, 2012 ▸) and *Mercury* (Macrae *et al.*, 2008 ▸); software used to prepare material for publication: *PLATON* (Spek, 2003 ▸) and *WinGX* (Farrugia, 2012 ▸).

## Supplementary Material

Crystal structure: contains datablock(s) global, I. DOI: 10.1107/S2056989015016709/su5203sup1.cif

Structure factors: contains datablock(s) I. DOI: 10.1107/S2056989015016709/su5203Isup2.hkl

Supporting information file. DOI: 10.1107/S2056989015016709/su5203Isup3.cml

**Fig. 1.**
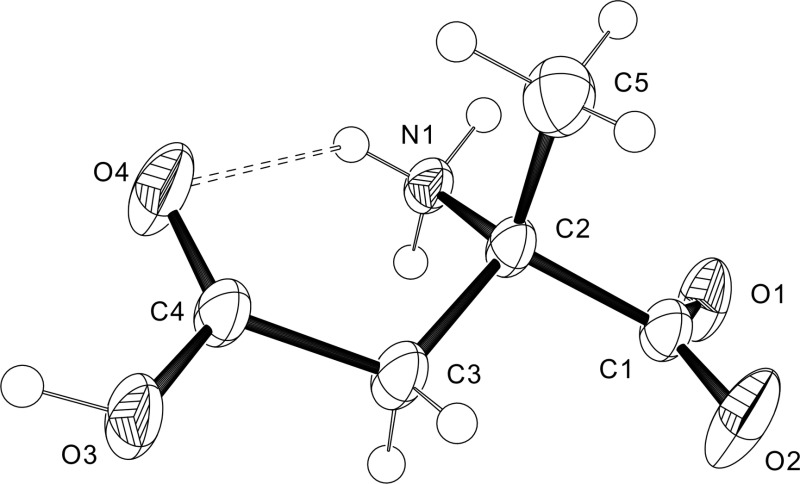
A view of the molecular structure of the title compound, with atom labelling. Displacement ellipsoids are drawn at the 50% probability level. The dashed line indicates the intramolecular N—H···O hydrogen bond (see Table 1).

. DOI: 10.1107/S2056989015016709/su5203fig1.tif
A view of the mol­ecular structure of the title compound, with atom labelling. Displacement ellipsoids are drawn at the 50% probability level. The dashed line indicates the intra­molecular N—H⋯O hydrogen bond (see Table 1).

**Fig. 2.**
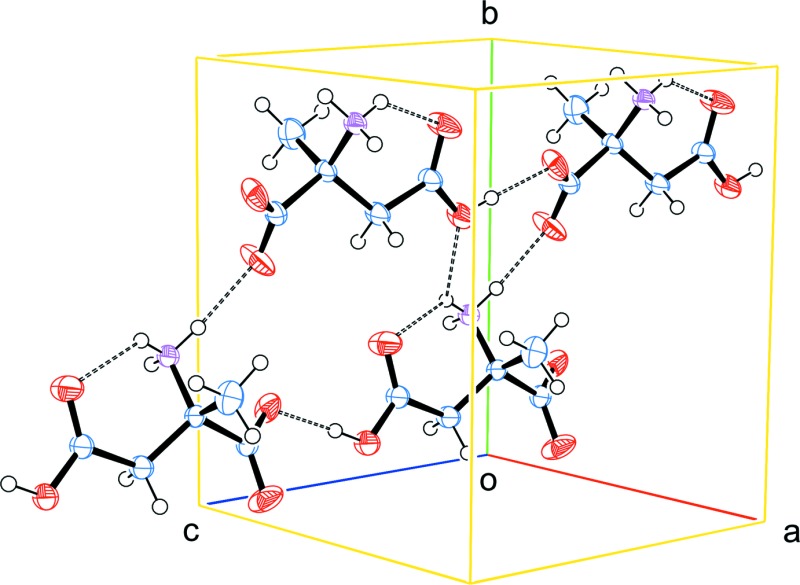
A partial view of the crystal packing of the title compound. Dashed lines indicate the O—H···O and N—H···O hydrogen bonds (see Table 1).

**Fig. 3.**
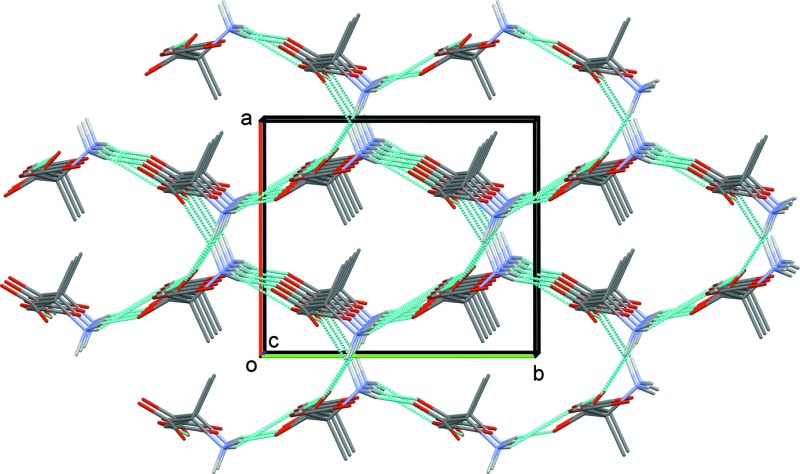
A view along the c axis of the crystal packing of the title compound. Dashed lines indicate the O—H···O and N—H···O hydrogen bonds (see Table 1), and C-bound H atoms have been omitted for clarity.

. DOI: 10.1107/S2056989015016709/su5203fig2.tif
A partial view of the crystal packing of the title compound. Dashed lines indicate the O—H⋯O and N—H⋯O hydrogen bonds (see Table 1).

c . DOI: 10.1107/S2056989015016709/su5203fig3.tif
A view along the *c* axis of the crystal packing of the title compound. Dashed lines indicate the O—H⋯O and N—H⋯O hydrogen bonds (see Table 1), and C-bound H atoms have been omitted for clarity.

CCDC reference: 1422827

Additional supporting information:  crystallographic information; 3D view; checkCIF report

## Figures and Tables

**Table 1:** Hydrogen-bond geometry (, )

*D*H*A*	*D*H	H*A*	*D* *A*	*D*H*A*
N1H7O4	0.79(4)	2.23(4)	2.798(3)	130(3)
O3H6O1^i^	0.84(4)	1.70(4)	2.543(2)	177(5)
N1H7O3^ii^	0.79(4)	2.53(4)	3.093(3)	130(3)
N1H8O2^iii^	0.86(3)	1.90(4)	2.754(3)	170(3)
N1H9O1^iv^	0.93(3)	1.93(4)	2.844(3)	168(4)
C3H3*B*O4^v^	0.97	2.52	3.279(4)	135

Symmetry codes: (i) 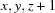; (ii) 
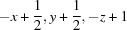
; (iii) 
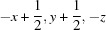
; (iv) 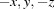; (v) 
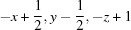
.

## supplementary crystallographic information

### S1. Comment

Solid-phase synthesis is now the accepted method to synthesis peptides, in which protected natural or non-natural amino acids are widely used; for example, 2-methyl­aspartic acid (MeASP) a non-natural amino acid. It has attracted attention as a substrate analog of aspartate amino­transferase (EC 2.6.1.1), and acts as a competitive inhibitor in the external aldimine (Delbaere *et al.*, 1989; Nobe *et al.*, 1998). Despite the biological and pharmaceutical inter­est, no crystal structures of MeASP derivatives have been reported except for the structure of DL-MeASP monohydrate (Brewer *et al.*, 2013).

In the title compound, Fig. 1, the succinic acid group has a *trans*-conformation [C1—C2—C3—C4 = -177.1 (2)°] *versus*. a *cis*-conformation [48.8 (4) °] in DL-MeASP. The carb­oxy group and the amino group make a hydrogen bonded half-chair S(6) ring motif (Table 1 and Fig. 1). The S(6) ring half-chair conformation and the *trans*-succinic acid arrangement are similar to the situation found in for DL-ASP (DLASPA03: Flaig *et al.* 1998).

In the crystal, molecules are linked by O—H···O hydrogen bonds, involving the succinic acid groups, to form C(7) chains along the *c* axis direction (Table 1 and Fig. 2). This is in contrast to the N—H···O hydrogen bonded C(5) chains observed in the crystal structure of DL-MeASP. The chains are linked by N—H···O and C—H···O hydrogen bonds forming sheets parallel to the *bc* plane. Further N—H···O hydrogen bonds link the sheets to form a three-dimensional framework (Table 1 and Fig. 3). The methyl groups are surrounded by the hydro­philic planes and make a columnar structure (Fig. 3).

### S2. Synthesis and crystallization

The title compound was purchased from Nagase-Sangyo Co. Ltd. The absolute configuration could not be established by anomalous-dispersion effects. The (*S*) enanti­omer has been chosen by referring the sign of known polarity in the synthetic procedure (Terashima *et al.*, 1966). Rod-like colourless crystals of the title compound were obtained by vapour-phase diffusion of an ethanol-chloro­form mixture at room temperature.

### S3. Refinement

Crystal data, data collection and structure refinement details are summarized in Table 2. All the H atoms were located in difference Fourier maps. The NH2 and OH H atoms were freely refined. The C-bound H atoms were included in calculated positions and treated as riding atoms: C–H = 0.96-0.97 Å with *U*_iso_(H) = 1.5U_eq_(C) for methyl H atoms and 1.2U_eq_(C) for other H atoms.

### Crystal data

**Table:**

C_5_H_9_NO_4_	*F*(000) = 312
*M**_r_* = 147.13	*D*_x_ = 1.508 Mg m^−^^3^
Monoclinic, *C*2	Cu *K*α radiation, λ = 1.54178 Å
Hall symbol: C 2y	Cell parameters from 25 reflections
*a* = 8.3398 (12) Å	θ = 20–28°
*b* = 9.6725 (10) Å	µ = 1.14 mm^−^^1^
*c* = 8.0671 (10) Å	*T* = 297 K
β = 95.175 (5)°	Rod, colorless
*V* = 648.09 (14) Å^3^	0.4 × 0.2 × 0.2 mm
*Z* = 4	

### Data collection

**Table:**

Enraf–Nonius CAD-4 diffractometer	699 reflections with *I* > 2σ(*I*)
Radiation source: sealed X-ray tube	*R*_int_ = 0.019
Graphite monochromator	θ_max_ = 74.0°, θ_min_ = 5.5°
ω/2θ scans	*h* = −10→1
Absorption correction: ψ scan (North *et al.*, 1968)	*k* = −12→0
*T*_min_ = 0.76, *T*_max_ = 0.81	*l* = −10→10
843 measured reflections	3 standard reflections every 300 reflections
700 independent reflections	intensity decay: none

### Refinement

**Table:**

Refinement on *F*^2^	Secondary atom site location: difference Fourier map
Least-squares matrix: full	Hydrogen site location: mixed
*R*[*F*^2^ > 2σ(*F*^2^)] = 0.034	H atoms treated by a mixture of independent and constrained refinement
*wR*(*F*^2^) = 0.096	*w* = 1/[σ^2^(*F*_o_^2^) + (0.0559*P*)^2^ + 0.2563*P*] where *P* = (*F*_o_^2^ + 2*F*_c_^2^)/3
*S* = 1.27	(Δ/σ)_max_ < 0.001
700 reflections	Δρ_max_ = 0.29 e Å^−^^3^
109 parameters	Δρ_min_ = −0.21 e Å^−^^3^
2 restraints	Extinction correction: *SHELXL2014* (Sheldrick, 2014), Fc^*^=kFc[1+0.001xFc^2^λ^3^/sin(2θ)]^-1/4^
Primary atom site location: structure-invariant direct methods	Extinction coefficient: 0.045 (4)

### Special details

**Table:**

Geometry. All e.s.d.'s (except the e.s.d. in the dihedral angle between two l.s. planes) are estimated using the full covariance matrix. The cell e.s.d.'s are taken into account individually in the estimation of e.s.d.'s in distances, angles and torsion angles; correlations between e.s.d.'s in cell parameters are only used when they are defined by crystal symmetry. An approximate (isotropic) treatment of cell e.s.d.'s is used for estimating e.s.d.'s involving l.s. planes.

### Fractional atomic coordinates and isotropic or equivalent isotropic displacement parameters (Å^2^)

**Table:**

	*x*	*y*	*z*	*U*_iso_*/*U*_eq_	
H8	0.155 (3)	0.434 (4)	0.122 (4)	0.026 (7)*	
H7	0.126 (4)	0.416 (4)	0.275 (5)	0.040 (9)*	
H6	0.207 (5)	0.170 (5)	0.725 (4)	0.071 (14)*	
H9	0.022 (4)	0.340 (4)	0.164 (4)	0.040 (9)*	
C1	0.2417 (3)	0.1802 (3)	0.0388 (3)	0.0273 (5)	
C2	0.2485 (3)	0.2608 (2)	0.2046 (3)	0.0238 (5)	
C3	0.2132 (3)	0.1603 (3)	0.3436 (3)	0.0305 (6)	
H3A	0.1108	0.1156	0.3124	0.037*	
H3B	0.2954	0.0891	0.3512	0.037*	
C4	0.2069 (3)	0.2244 (3)	0.5137 (3)	0.0284 (6)	
C5	0.4148 (3)	0.3267 (4)	0.2343 (4)	0.0386 (7)	
H5A	0.4277	0.3947	0.1498	0.058*	
H5B	0.4959	0.2567	0.2301	0.058*	
H5C	0.4254	0.3702	0.3417	0.058*	
N1	0.1250 (3)	0.3741 (2)	0.1916 (3)	0.0248 (5)	
O1	0.1677 (2)	0.2358 (2)	−0.0871 (2)	0.0378 (5)	
O2	0.3164 (3)	0.0705 (2)	0.0427 (3)	0.0525 (7)	
O3	0.2242 (3)	0.1339 (2)	0.6334 (2)	0.0392 (6)	
O4	0.1831 (4)	0.3463 (2)	0.5370 (2)	0.0554 (7)	

### Atomic displacement parameters (Å^2^)

**Table:**

	*U*^11^	*U*^22^	*U*^33^	*U*^12^	*U*^13^	*U*^23^
C1	0.0384 (11)	0.0263 (12)	0.0177 (10)	0.0010 (10)	0.0051 (8)	−0.0020 (9)
C2	0.0349 (10)	0.0220 (11)	0.0147 (10)	0.0029 (9)	0.0028 (8)	−0.0013 (8)
C3	0.0520 (14)	0.0239 (13)	0.0157 (10)	0.0039 (11)	0.0039 (9)	−0.0005 (9)
C4	0.0436 (13)	0.0252 (12)	0.0164 (10)	0.0015 (10)	0.0025 (9)	−0.0014 (9)
C5	0.0342 (12)	0.0476 (17)	0.0338 (13)	−0.0022 (12)	0.0020 (10)	−0.0065 (12)
N1	0.0368 (11)	0.0213 (10)	0.0164 (9)	0.0008 (8)	0.0036 (7)	−0.0007 (8)
O1	0.0510 (10)	0.0455 (11)	0.0167 (8)	0.0152 (9)	0.0011 (7)	−0.0039 (8)
O2	0.0930 (17)	0.0383 (13)	0.0257 (10)	0.0290 (13)	0.0023 (10)	−0.0082 (9)
O3	0.0721 (13)	0.0304 (10)	0.0162 (9)	0.0085 (9)	0.0096 (8)	0.0010 (8)
O4	0.118 (2)	0.0290 (12)	0.0200 (9)	0.0126 (12)	0.0102 (10)	−0.0024 (8)

### Geometric parameters (Å, º)

**Table:**

C1—O2	1.229 (3)	C4—O4	1.213 (4)
C1—O1	1.261 (3)	C4—O3	1.301 (3)
C1—C2	1.545 (3)	C5—H5A	0.9600
C2—N1	1.502 (3)	C5—H5B	0.9600
C2—C5	1.525 (3)	C5—H5C	0.9600
C2—C3	1.532 (3)	N1—H8	0.86 (4)
C3—C4	1.511 (3)	N1—H7	0.78 (4)
C3—H3A	0.9700	N1—H9	0.93 (4)
C3—H3B	0.9700	O3—H6	0.84 (2)
			
O2—C1—O1	126.8 (2)	O4—C4—C3	124.0 (2)
O2—C1—C2	115.7 (2)	O3—C4—C3	112.8 (2)
O1—C1—C2	117.3 (2)	C2—C5—H5A	109.5
N1—C2—C5	108.3 (2)	C2—C5—H5B	109.5
N1—C2—C3	109.79 (18)	H5A—C5—H5B	109.5
C5—C2—C3	112.5 (2)	C2—C5—H5C	109.5
N1—C2—C1	109.67 (18)	H5A—C5—H5C	109.5
C5—C2—C1	107.98 (18)	H5B—C5—H5C	109.5
C3—C2—C1	108.60 (19)	C2—N1—H8	107 (2)
C4—C3—C2	115.4 (2)	C2—N1—H7	112 (3)
C4—C3—H3A	108.4	H8—N1—H7	104 (3)
C2—C3—H3A	108.4	C2—N1—H9	112 (3)
C4—C3—H3B	108.4	H8—N1—H9	113 (3)
C2—C3—H3B	108.4	H7—N1—H9	109 (3)
H3A—C3—H3B	107.5	C4—O3—H6	111 (4)
O4—C4—O3	123.2 (2)		

### Hydrogen-bond geometry (Å, º)

**Table:**

*D*—H···*A*	*D*—H	H···*A*	*D*···*A*	*D*—H···*A*
N1—H7···O4	0.79 (4)	2.23 (4)	2.798 (3)	130 (3)
O3—H6···O1^i^	0.84 (4)	1.70 (4)	2.543 (2)	177 (5)
N1—H7···O3^ii^	0.79 (4)	2.53 (4)	3.093 (3)	130 (3)
N1—H8···O2^iii^	0.86 (3)	1.90 (4)	2.754 (3)	170 (3)
N1—H9···O1^iv^	0.93 (3)	1.93 (4)	2.844 (3)	168 (4)
C3—H3*B*···O4^v^	0.97	2.52	3.279 (4)	135

Symmetry codes: (i) *x*, *y*, *z*+1; (ii) −*x*+1/2, *y*+1/2, −*z*+1; (iii) −*x*+1/2, *y*+1/2, −*z*; (iv) −*x*, *y*, −*z*; (v) −*x*+1/2, *y*−1/2, −*z*+1.

## References

[bb1] Brewer, G., Burton, A. S., Dworkin, J. P. & Butcher, R. J. (2013). *Acta Cryst.* E**69**, o1856–o1857. 10.1107/S1600536813032170 PMC3885094 24454270

[bb2] Delbaere, L. T., Kallen, J., Markovic-Housley, Z., Khomutov, A. R., Khomutov, R. M., Karpeisky, M. Y. & Jansonius, J. N. (1989). *Biochimie*, **71**, 449–459. 10.1016/0300-9084(89)90175-2 2503050

[bb3] Derricott, C. & Trotter, J. (1979). *Acta Cryst.* B**35**, 2230–2232.

[bb4] Enraf–Nonius (1989). *CAD-4 Software*. Enraf–Nonius, Delft, The Netherlands.

[bb5] Farrugia, L. J. (2012). *J. Appl. Cryst.* **45**, 849–854.

[bb6] Flaig, R., Koritsanszky, T., Zobel, D. & Luger, P. (1998). *J. Am. Chem. Soc.* **120**, 2227–2238.

[bb7] Harms, K. & Wocadlo, S. (1995). *XCAD4*. University of Marburg, Germany.

[bb8] Macrae, C. F., Bruno, I. J., Chisholm, J. A., Edgington, P. R., McCabe, P., Pidcock, E., Rodriguez-Monge, L., Taylor, R., van de Streek, J. & Wood, P. A. (2008). *J. Appl. Cryst.* **41**, 466–470.

[bb9] Nobe, Y., Kawaguchi, S., Ura, H., Nakai, T., Hirotsu, K., Kato, R. & Kuramitsu, S. (1998). *J. Biol. Chem.* **273**, 29554–29564. 10.1074/jbc.273.45.29554 9792664

[bb10] North, A. C. T., Phillips, D. C. & Mathews, F. S. (1968). *Acta Cryst.* A**24**, 351–359.

[bb11] Pfeiffer, P. & Heinrich, E. (1936). *J. Prakt. Chem.* **146**, 105–112.

[bb12] Sheldrick, G. M. (2008). *Acta Cryst.* A**64**, 112–122. 10.1107/S0108767307043930 18156677

[bb13] Sheldrick, G. M. (2015). *Acta Cryst.* C**71**, 3–8.

[bb14] Spek, A. L. (2003). *J. Appl. Cryst.* **36**, 7–13.

[bb15] Terashima, S., Achiwa, K. & Yamada, S. (1966). *Chem. Pharm. Bull.* **14**, 572–578. 10.1248/cpb.14.572 5964621

