# Supplementary figures and images for: Crystal structure of (S)-2-amino-2-methyl­succinic acid
Source: Acta Crystallogr E Crystallogr Commun. 2015 Sep 12;71(Pt 10):o731–2. doi: 10.1107/S2056989015016709 (PMC4647375; doi:10.1107/S2056989015016709)

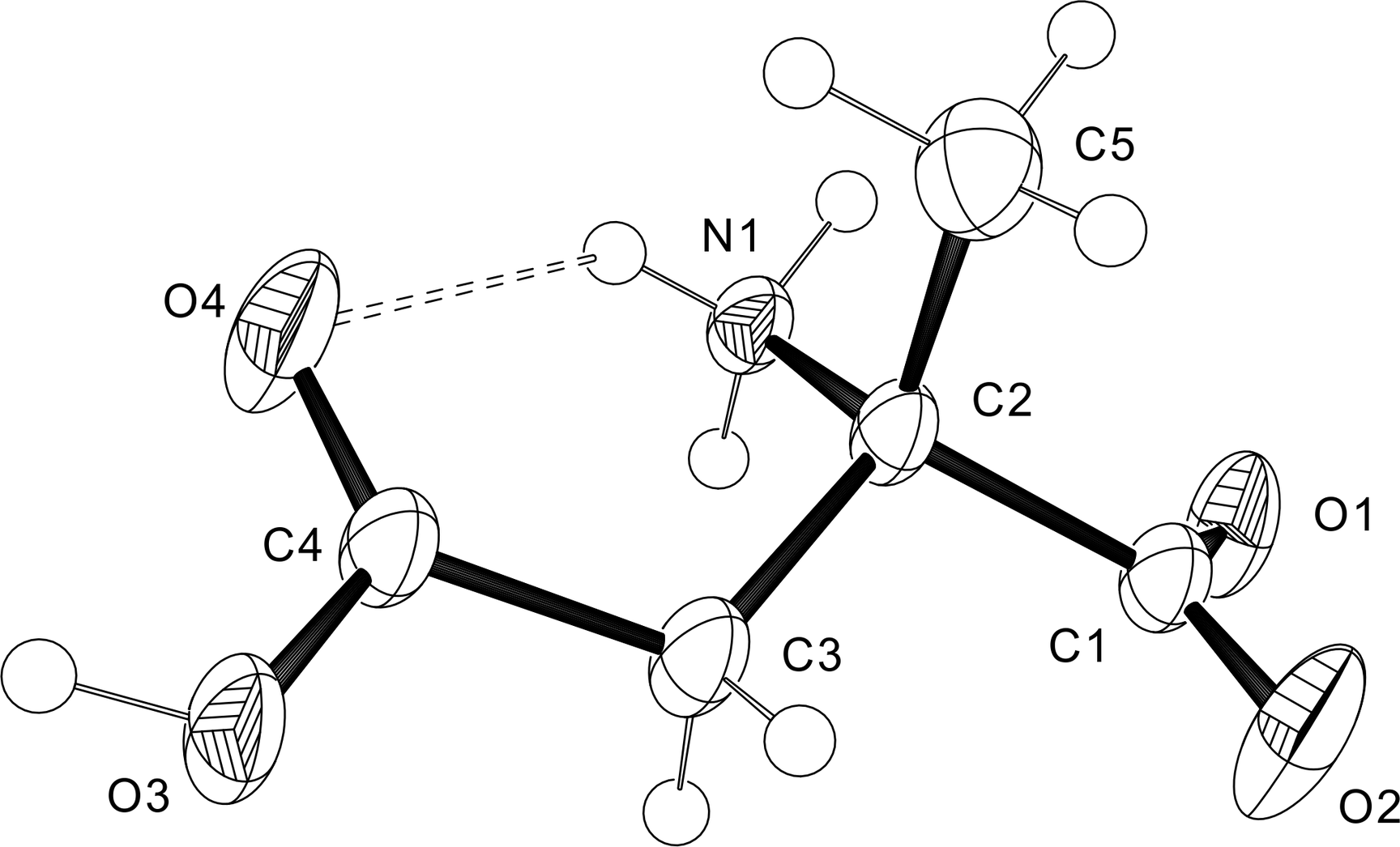

Supplement: Supplementary file 4 [file e-71-0o731-fig1.tif]

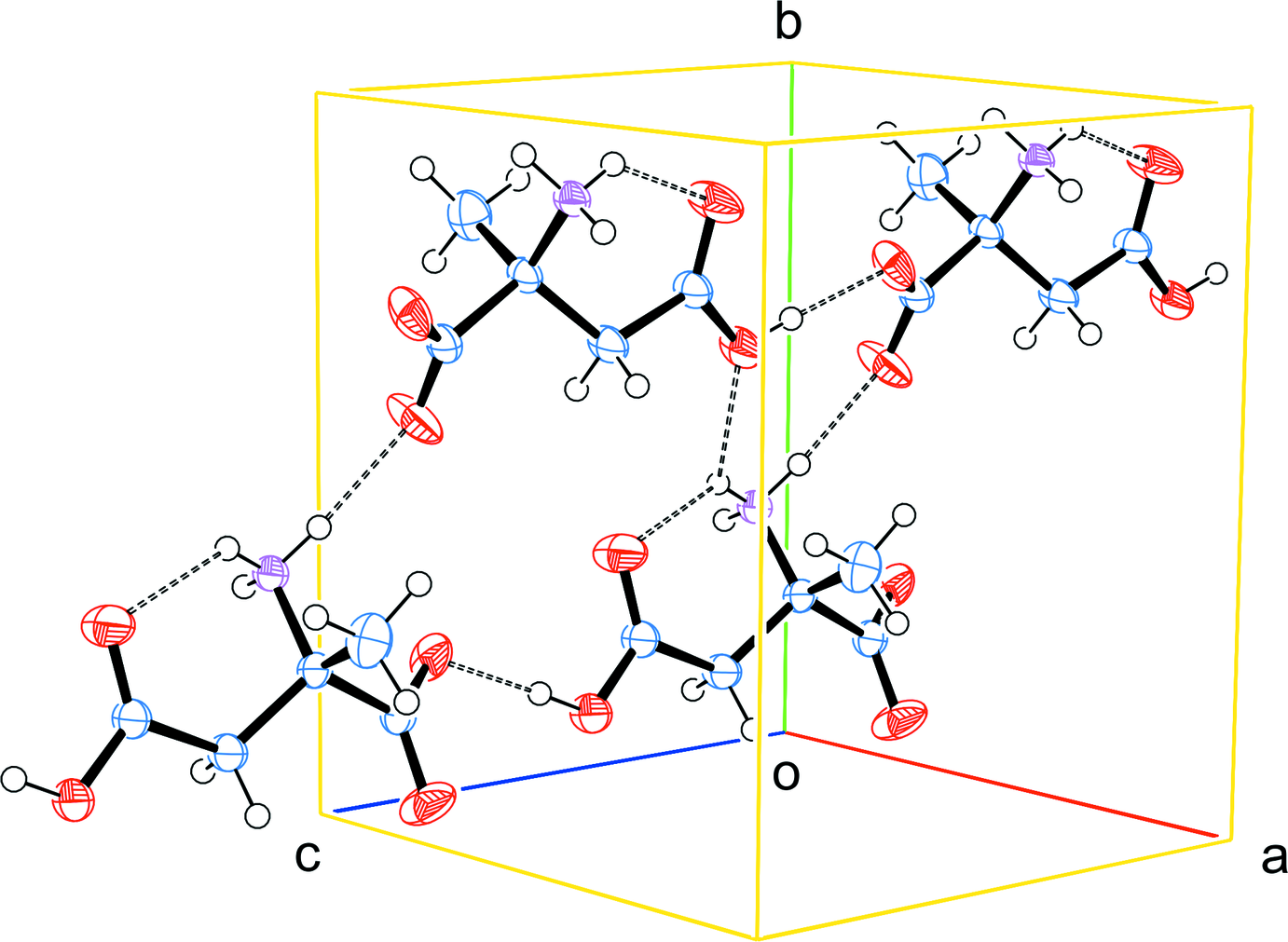

Supplement: Supplementary file 5 [file e-71-0o731-fig2.tif]

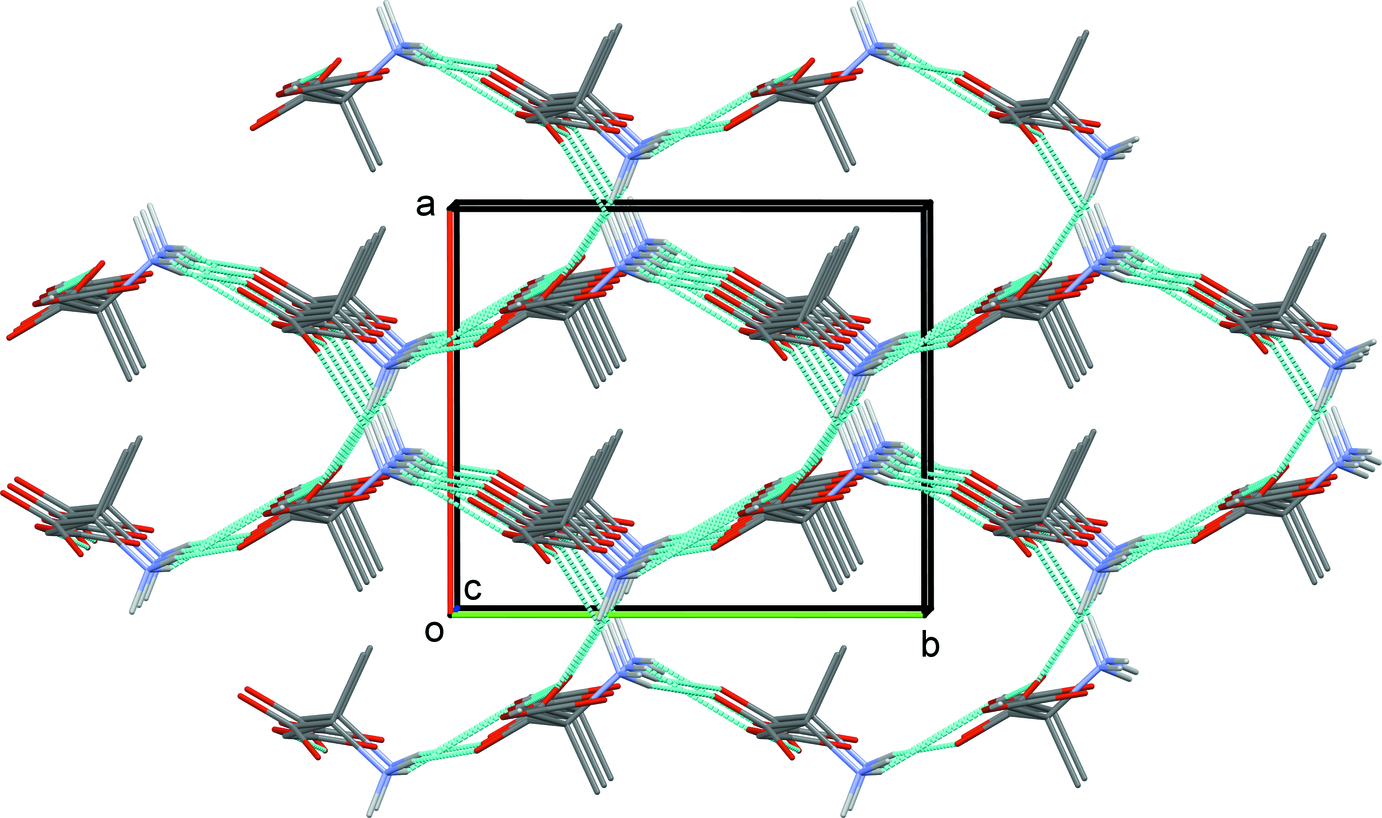

Supplement: Supplementary file 6 [file e-71-0o731-fig3.tif]
